# [Corrigendum] miR-185 enhances the inhibition of proliferation and migration induced by ionizing radiation in melanoma

**DOI:** 10.3892/ol.2026.15480

**Published:** 2026-02-04

**Authors:** Jinpeng He, Ning Tian, Yanli Yang, Liangliang Jin, Xiu Feng, Junrui Hua, Sulan Lin, Bing Wang, He Li, Jufang Wang

Oncol Lett 13: 2442–2448, 2017; DOI: 10.3892/ol.2017.5699

Following the publication of the above article, an interested reader drew to the authors’ attention that there appeared to be an overlapping section of data associated with the ‘IR PN’ and ‘IR P185’ data panels shown in Fig. 3C for the H&E staining experiments/row of data on p. 2446, such that data which were intended to show the results of different experiments had apparently been derived from the same original source.

Upon assessing their data, the authors realized that the data shown for the IR P185 experiment/data panel had inadvertently been included incorrectly in this figure. The revised version of Fig. 3, now showing the correct data for the IR P185 experiment in Fig. 3C, is shown on the next page. The authors regret that this error went unnoticed in the published version of this figure, although this did not grossly effect with the results or the conclusions reported in this article. All the authors agree with the publication of this Corrigendum, and thank the Editor of *Oncology Letters* for granting them the opportunity to publish this; furthermore, they apologize to the readership for any inconvenience caused.

## Figures and Tables

**Figure 3.**
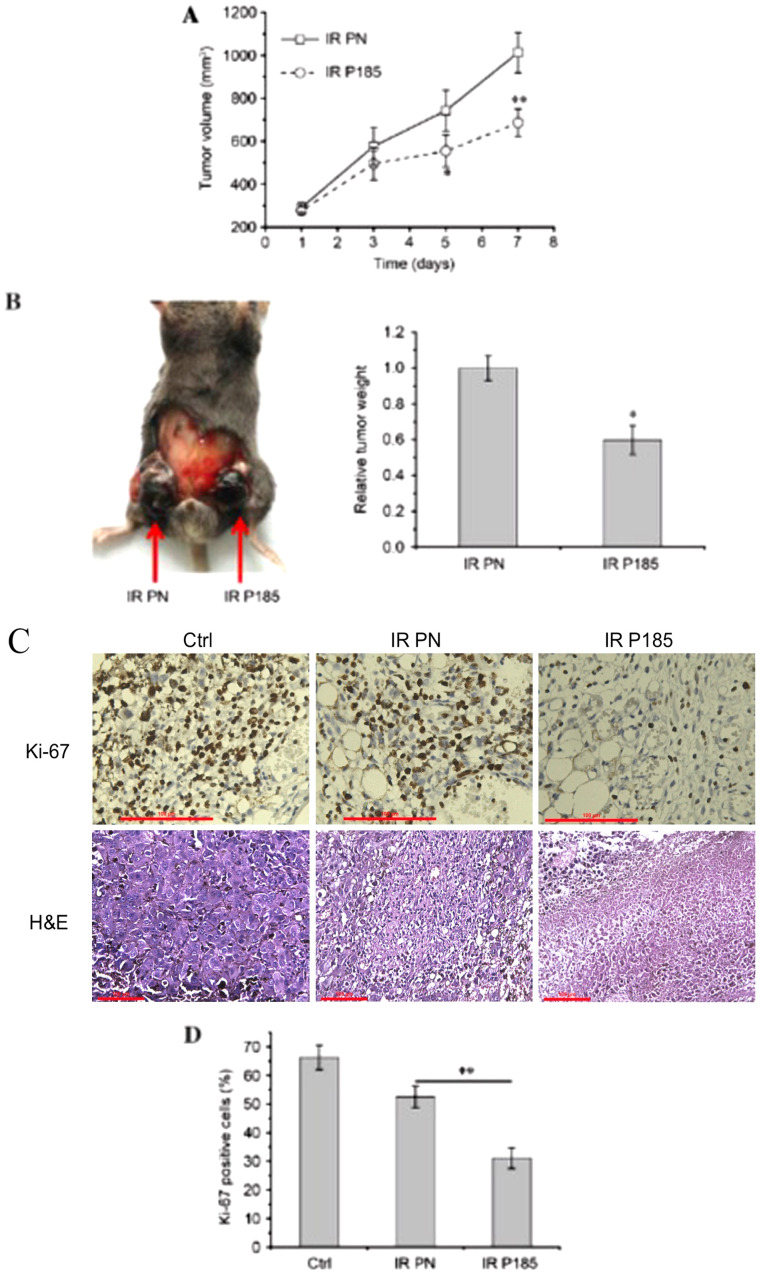
Effect of miR-185 on tumor radiotherapy outcome. B16 cells were injected subcutaneously into the groins of C57/BL6 mice. Following formation of tumors, mice (n=9) were selected at random on day 1. miRNA duplex were transfected into tumors on days 1, 3 and 5, whereas tumors were locally exposed to 4 Gy of X-rays on days 2, 4 and 6 independently. (A) Change in tumor volume over the treatment period. (B) Representative mouse with tumors captured on day 8 and relative tumor weight (IR P185 vs. IR PN). (C) Representative images of immunohistochemistry for Ki-67 (magnification, ×400) and H&E staining (magnification, ×200) in tumor tissues. (D) The allograft tumors removed from C57/BL6 mice were analyzed using immunohistochemistry for expression of Ki-67. *P<0.05, **P<0.01 vs. IR PN. Scale bars, 100 µm. miR-185, miRNA 185; miRNA, microRNA; IR, ionizing radiation; PN, pre-neg-transfected cells; P185, pre-miR-185-transfected cells; Ctrl, control; Ki-67, proliferation marker protein Ki-67; H&E, hematoxylin and eosin.

